# Carbon ion irradiation withstands cancer stem cells' migration/invasion process in Head and Neck Squamous Cell Carcinoma (HNSCC)

**DOI:** 10.18632/oncotarget.10281

**Published:** 2016-06-24

**Authors:** Coralie Moncharmont, Jean-Baptiste Guy, Anne-Sophie Wozny, Marion Gilormini, Priscilla Battiston-Montagne, Dominique Ardail, Michael Beuve, Gersende Alphonse, Xavier Simoëns, Chloé Rancoule, Claire Rodriguez-Lafrasse, Nicolas Magné

**Affiliations:** ^1^ Université Lyon 1, Faculté de Médecine-Lyon-Sud, Oullins, 69921, France; ^2^ Laboratoire de Radiobiologie Cellulaire et Moléculaire, Institut de Physique Nucléaire de Lyon, IPNL, Villeurbanne, 69622, France; ^3^ Département de Radiothérapie, Institut de Cancérologie de la Loire - Lucien Neuwirth, St Priest en Jarez, 42270, France; ^4^ Hospices Civils de Lyon, Lyon, 69229, France; ^5^ Institut de Physique Nucléaire de Lyon, IPNL, Villeurbanne, 69622, France; ^6^ Département de Pharmacologie Clinique et d'Innovation, Institut de Cancérologie de la Loire - Lucien Neuwirth, St Priest en Jarez, 42270, France

**Keywords:** carbon ion irradiation, cancer stem cells, migration, invasion, HNSCC

## Abstract

Cancer Stem Cells (CSCs) in Head and Neck Squamous Cell Carcinoma (HNSCC) have extremely aggressive profile (high migratory and invasive potential). These characteristics can explain their resistance to conventional treatment. Efficacy of photon and carbon ion irradiation with addition of cetuximab (5 nM) is studied on clonogenic death, migration and invasion of two HNSCC populations: SQ20B and SQ20B/CSCs. SQ20B express E-cadherin and overexpress EGFR while SQ20B/CSCs express N-cadherin and low EGFR. Cetuximab strongly inhibits SQ20B proliferation but has no effect on SQ20B/CSCs. 2 Gy photon irradiation enhances migration and invasiveness in both populations (*p* < 0.05), while cetuximab only stops SQ20B migration (*p* < 0.005). Carbon irradiation significantly inhibits invasion in both populations (*p* < 0.05), and the association with cetuximab significantly inhibits invasion in both populations (*p* < 0.005). These results highlight CSCs characteristics: EGFR^Low^, cetuximab-resistant, and highly migratory. Carbon ion irradiation appears to be a very promising therapeutic modality counteracting migration/invasion process in both parental cells and CSCs in contrast to photon irradiation.

## INTRODUCTION

In Head and Neck Squamous Cell Carcinoma (HNSCC), about two-thirds of patients present an advanced stage disease at diagnosis, either due to a regional lymph node involvement, and/or a large tumor size [1]. Aggressive treatment protocols, such as surgery, followed by radiation therapy (RT) with or without concomitant chemotherapy (CT) [2] are used in this curative situation. Cisplatin based chemoradiotherapy remains one of the great standard of care in this situation, as the same matter as carboplatin + 5-Fluorouracil or cetuximab in combination. However cisplatin scheme is related to long-term severe side effects or premature CT disruption. Suboptimal CT dosing may impact negatively on disease-free survival [3]. In parallel, targeted therapies now take a major place in anti-cancer treatment, particularly in HNSCC where Epidermal Growth Factor Receptor (EGFR)'s overexpression, is associated with a poor prognosis [4]. Cetuximab, a mouse-human chimeric monoclonal antibody directed against EGFR, significantly improves locoregional control, progression-free survival, and overall survival when used concomitantly with RT [5]. If targeted therapies are increasingly used, cetuximab is the only drug that have proved efficacy in association with radiation therapy. But whatever the therapeutic management, HNSCC is still associated with a high rate of recurrences [6]. The relative failure of conventional therapies led to the emergence of hadrontherapy in head and neck cancer management. Since two decades, clinical trials with carbon ion hadrontherapy assessed the benefit to treat photon resistant cancers, with an acceptable toxicity [7]. Carbon ion radiation is efficient in some types of head and neck cancer such as reported in the phase II trial published by Mizoe et al. [8]. Ongoing clinical trials are combining photon and carbon radiations with cetuximab in oro-pharynx or larynx carcinomas [9].

However, metastatic disease remains the leading cause of death in cancer [10]. If carbon ion irradiation proved benefit in local control, metastasis recurrences were not decreased. Cell migration and invasion is a substantial step of the metastatic phenomenon, several *in vitro* studies demonstrated that cells' invasion/migration could be increased by photon radiation [11–13]. A subpopulation of cancer cells, the cancer stem cells (CSCs), has shown a high migratory potential [14]. These cells are present in HNSCC [15], and overexpress CD44 and ALDH proteins, which are now considered as a HNSCC CSCs' marker [16]. Up to now, data on HNSCC CSCs' invasiveness are scarce. Data on migration are of particular interest on cells exposed to cetuximab and photon or carbon ion radiation.

Thus, the aim of the present work is to investigate, *in vitro*, the impact of a combined treatment associating photon or carbon radiation plus cetuximab, on proliferation and invasiveness, for both, parental and stem cells subpopulations.

## RESULTS

### Cell proliferation and survival after treatment by photon radiation

**Figure 1:**
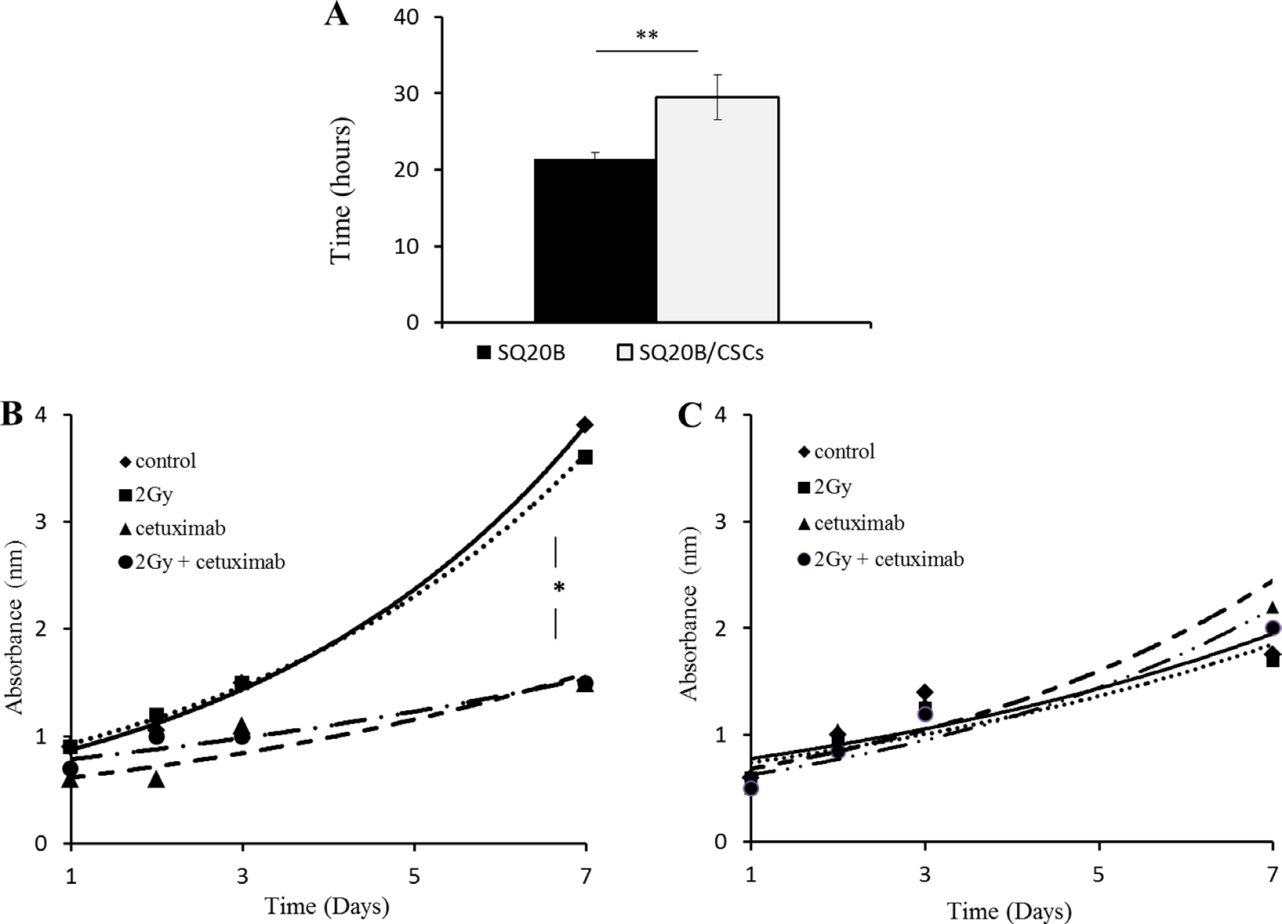
(**A**) Doubling time of parental SQ20B cells and its subpopulation SQ20B/CSCs in basal conditions. Effect of 5 nM cetuximab and 2 Gy photon radiation (IR) on proliferation of (**B**) SQ20B cells and its subpopulation (**C**) SQ20B/CSCs. Proliferation was measured with absorbance during 7 days. *p < 0.05, **p < 0.01.

In basal conditions, the SQ20B parental cell lines have proliferated about 25% faster than the SQ20B/CSCs subpopulation (Figure 1A). The 2 Gy photon irradiation did not inhibit cell proliferation whether parental SQ20B or SQ20B/CSCs (Figure 1B). Both mono-treatment with cetuximab or combined treatment reduced SQ20B proliferation but not SQ20B/CSCs proliferation. The calculated SF2 of SQ20B was significantly decreased with cetuximab (0.81 vs 0.62 without and with cetuximab, respectively, *p* = 0.007) in contrast to SQ20B/CSCs (0.77 vs 0.73, with and without cetuximab respectively *p* = 0.62).

### Expression of EGFR and downstream signaling

**Figure 2:**
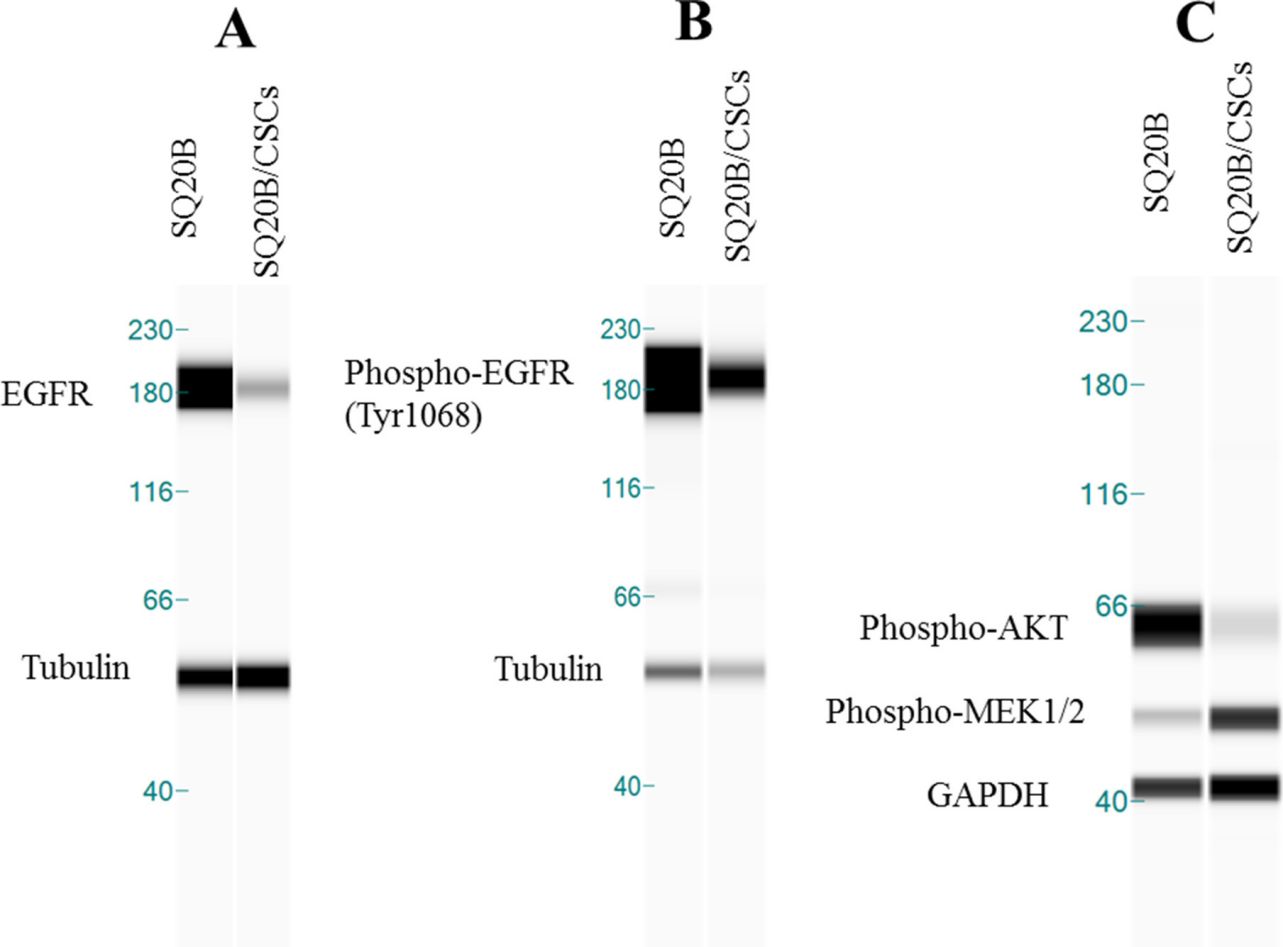
(**A**) EGFR basal expression in SQ20B cells and its subpopulation SQ20B/CSCs. Protein expression analysis was done with WES™*. (**B**) Phospho-EGFR of Tyr1068 in basal condition in SQ20B cells and its subpopulation SQ20B/CSCs. Tubulin was used as a reference protein. (**C**) Phospho-AKT (Ser 473) and Phospho-MEK1/2 (Ser217/221) in basal condition in SQ20B cells and its subpopulation SQ20B/CSCs. GAPDH was used as a reference protein. *WES is a simple western technique using an automated capillary-based size sorting system.

EGFR in SQ20B/CSCs subpopulation was under-expressed compared with SQ20B cells. This result was confirmed with conventional western blotting experiments (data not shown). This receptor was phosphorylated on Tyrosine 1068 in basal condition in both, SQ20B cells and SQ20B/CSCs subpopulation (Figure 2A, 2B). In parallel, SQ20B cells express phospho-AKT while SQ20B/CSCs express phospho-MEK1/2 (Figure 2C).

### Cell invasion/migration abilities and Epithelio-Mesenchymal Transition (EMT) markers

**Figure 3:**
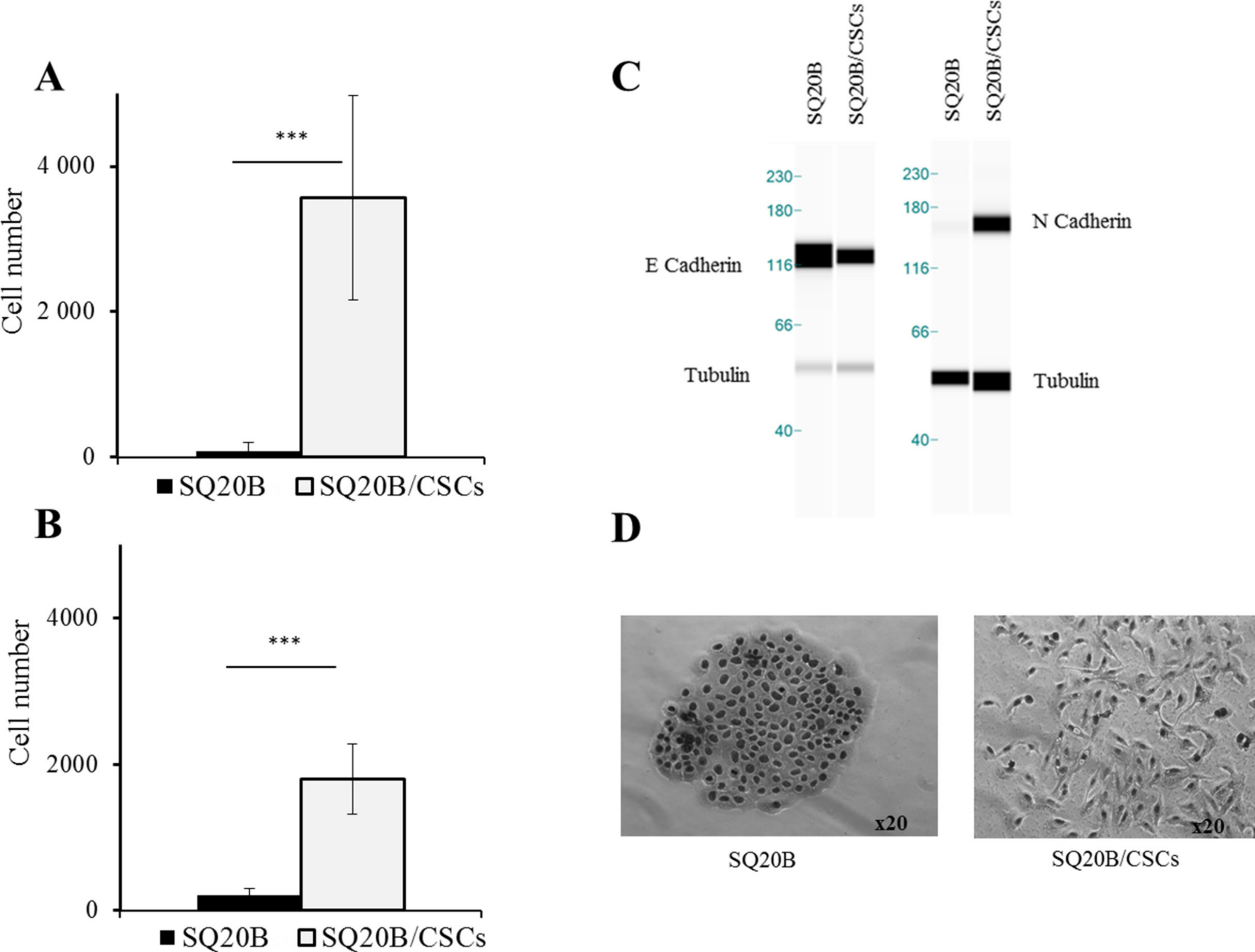
(**A**) Migration and (**B**) invasion abilities of SQ20B cells and their SQ20B/CSCs subpopulation. 30000 cells were put in each transwell, Cells that were below the membrane were counted. ***p < 0.005. EMT phenotype was characterized with E-cadherin and N-cadherin expression (**C**) with WES™* and cellular morphology in optical microscopy (x20) (**D**). *WES is a simple western technique using an automated capillary-based size sorting system.

SQ20B/CSCs migration and invasion capacities were higher to SQ20B parental cells in basal conditions (*p* < 0.005) (Figure 3A, 3B). This is related to their mesenchymal phenotype, SQ20B/CSCs exhibiting a high N-cadherin expression and a low E-cadherin expression. At the contrary, SQ20B parental cells show an epithelial phenotype with many cell-cell junctions and a high E-cadherin expression (Figure 3C, 3D).

### Effect of photon irradiation and/or cetuximab on cell migration/invasion

**Figure 4:**
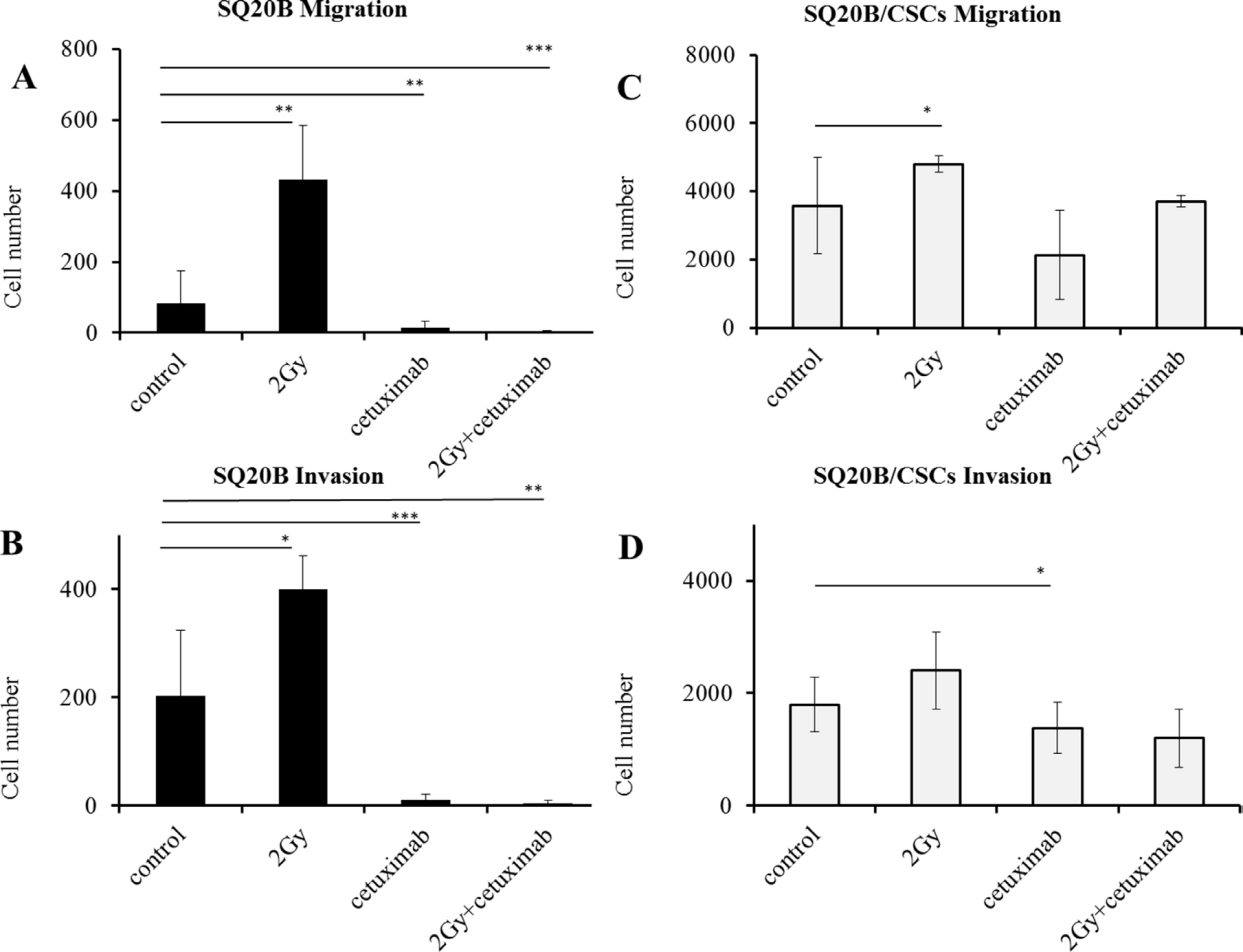
Influence of photon radiation and/or cetuximab on migration and invasion abilities of SQ20B parental cells and their SQ20B/CSCs subpopulation (**A**) SQ20B Migration; (**B**) SQ20B Invasion; (**C**) SQ20B/CSCs Migration; (**D**) SQ20B/CSCs Invasion. 30000 cells were put in each transwell, Cetuximab concentration was 5 nM. *p < 0.05, **p < 0.01, ***p < 0.005.

Migration and invasion were significantly enhanced by a 2 Gy irradiation in SQ20B cells (*p* < 0.01 and *p* < 0.05). Cetuximab reduced both migration and invasion (*p* < 0.01 and *p* < 0.005), even more when it is associated with photon radiation (*p* < 0.005 and *p* < 0.01) (Figure 4A, 4B). The SQ20B/CSCs subpopulation, migrated and invaded in Matrigel ten times more than SQ20B cells (Figure 4C, 4D). Radiation enhanced slightly more SQ20B/CSCs migration (*p* < 0.05) but had no effect on invasion. Cetuximab weakly reduced their invasion (*p* < 0.05) whereas its association with photon radiation did not provide benefit.

### Effect of Carbon ion irradiation and/or cetuximab on cell migration/invasion

**Figure 5:**
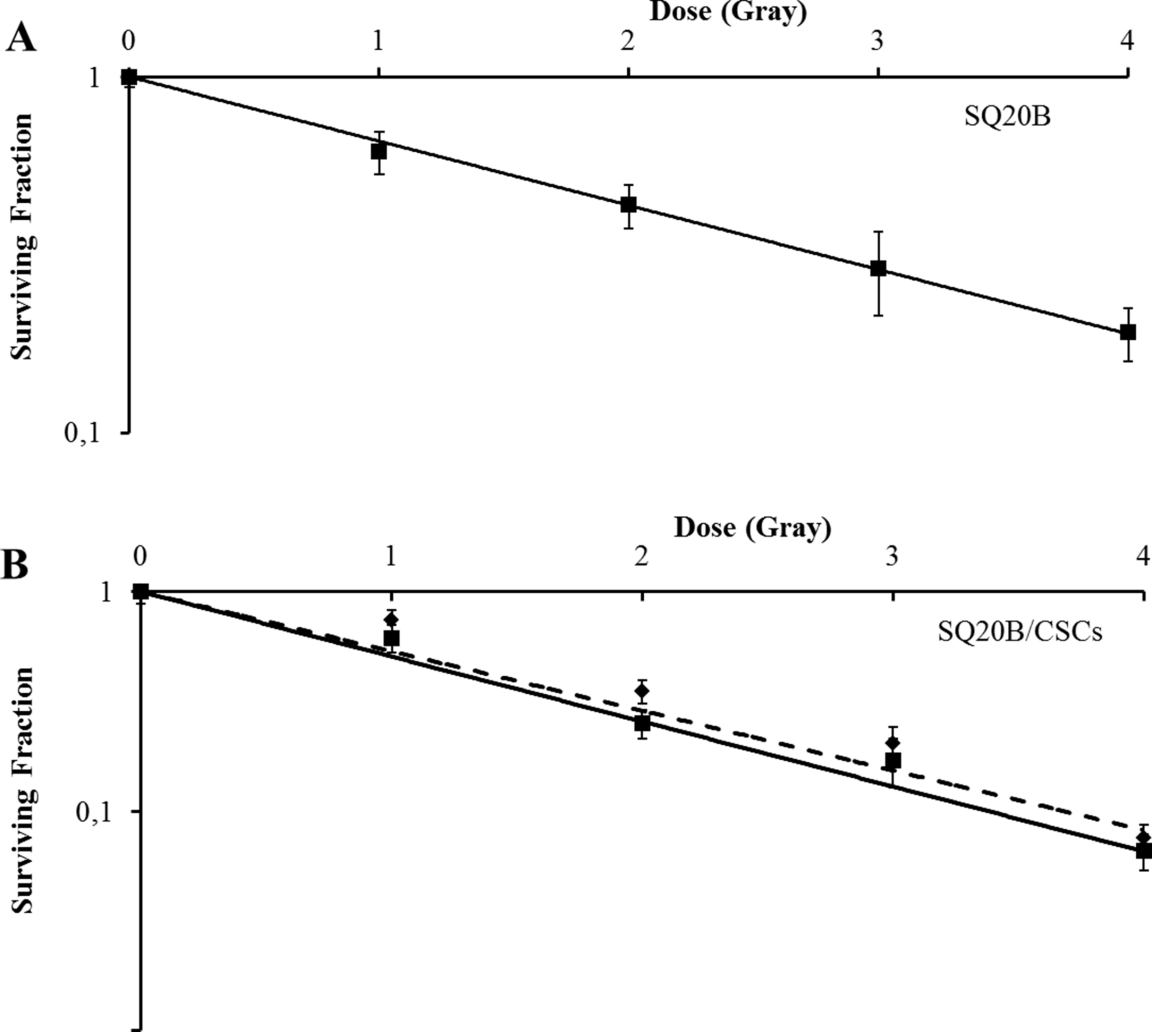
Survival curves of (**A**) SQ20B and (**B**) SQ20B/CSCs after cetuximab and/or carbon ion radiation exposition (full line: without cetuximab/dotted line: with 5 nM cetuximab). No cell colony was obtained when with treated SQ20B cells with cetuximab plus carbon ion radiation.

Carbon ion radiation reduced survival fraction of SQ20B and SQ20B/CSCs, with a relative biologic effectiveness (RBE) at 10% survival of 1.6 and 1.8 respectively. Interestingly, the association of cetuximab with carbon ion radiation was highly cytotoxic for SQ20B cells, seeing as no colony of more than 64 cells appeared at 2 Gy (Figure 5A) whereas it had no effect on the survival fraction of SQ20B/CSCs (Figure 5B).

**Figure 6:**
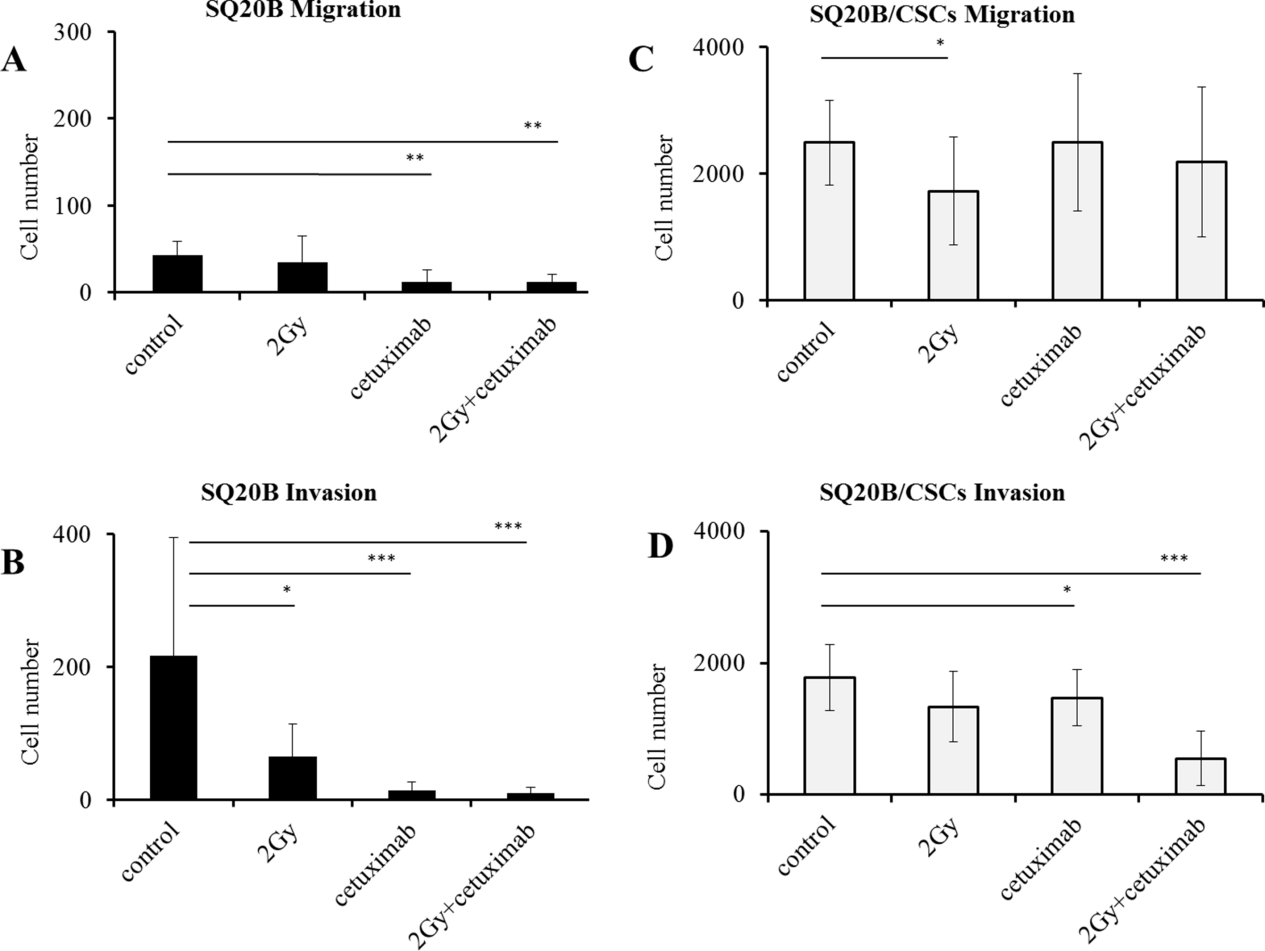
Influence of 2 Gy carbon ion radiation and/or 5 nM cetuximab on migration and invasion abilities of SQ20B parental cells and their SQ20B/CSCs subpopulation (**A**) SQ20B Migration; (**B**) SQ20B Invasion; (**C**) SQ20B/CSCs Migration; (**D**) SQ20B/CSCs Invasion. 30000 cells were put in each transwell, Cetuximab concentration was 5 nM. *p < 0.05, **p < 0.01, ***p < 0.005.

Increased migration and invasion of SQ20B and SQ20B/CSCs was not observed after carbon ion exposure (Figure 6A, 6B). The association of carbon ion radiation with cetuximab fully inhibited migration and invasion in SQ20B cells (*p* < 0.01 and *p* < 0,005). Migration of SQ20B/CSCs was significantly decreased by carbon ion radiation (*p* < 0.05) (Figure 6C). A decrease of the invasion in SQ20B/CSCs subpopulation was observed after the combined treatments (*p* < 0.005) (Figure 6D).

## DISCUSSION

This study identifies a subpopulation of HNSCC cancer stem cells with a high invasive potential and resistant to conventional treatment. The SQ20B/CSCs population has extremely high aggressive characteristics such as high migratory and invasive potential, associated with a mesenchymal phenotype. This study provides the first data on radiation efficacy (photon or carbon ions) on HNSCC CSCs migration and invasion process.

### EGFR expression and downstream signaling

In HNSCC, EGFR is overexpressed in 80–100% of the cases [17], and is a central hub of many signaling pathways such as Ras-MAPK and AKT-mTOR. EGFR is known to play a central role in stemness, particularly in colon [18, 19] and lung cancer stem cells [20, 21]. EGFR expression, either by binding [22] or immunohistochemistry (IHC) [23], appeared as an independent strong prognostic factor of locoregional failure in several clinical studies [24, 25]. Mutations in the EGFR tyrosine kinase domain appear to be rare in HNSCC [26, 27]. EGFRvIII [28] truncated mutants have been described in 42% of HNSCC patients [29] in one study and were associated with poorer prognosis and response to treatment compared to EGFR wild type. Yet, the absence of a reliable specific commercialized anti-EGFRvIII antibody makes generalization impossible. Moreover, the development of skin rash has been correlated with a clinical benefit in some tumors [30, 31] but this only indicates a potential benefit of continuing cetuximab or may suggest increasing the cetuximab dosage. However, it does not tell which patients should receive cetuximab at initiation of treatment. This aspect is of particular interest in our study, where cetuximab strongly inhibits SQ20B proliferation where EGFR is overexpressed in contrast to SQ20B/CSCs where no drug effect and a low EGFR expression are observed (Figures 1B, 1C–2A). If basal expression of EGFR is low in SQ20B/CSCs, its activation through the phosphorylation of 1068 Tyrosine is observed in both cell lines (Figure 2B). This activation leads to the PI3K-AKT-mTOR cascade and proliferation, but also to the MAPK cascade, through Grb2 protein which is the most important pathway activated by 1068 Tyrosine [32]. Interestingly, SQ20B parental cells express phospho-AKT protein, in contrast to SQ20B/CSCs cells which express phospho-MEK1/2. Activation of the MAPK cascade in SQ20B/CSCs can explain their motility capacities through EMT [33]. Constitutive activation of signaling pathways down-stream of EGFR by mutation or upregulation can promote survival [34]. For example, high level of activated Akt can occur downstream of EGFR inhibition through upstream-activated Src, Ras or mutated PTEN [35], amplification of the catalytic subunit of PI3K [36], or loss of the phosphatase and tensin homolog tumor suppressor protein [37]. By these results, we may explain the low sensitivity of SQ20B/CSCs to cetuximab, compared with the parental cells. These results fit with a recent publication on EGFR^Low^ head and neck cells [38]. In SQ20B/CSCs, receptor phosphorylation through homo or hetero-dimerization blocks cetuximab binding, and its therapeutic effect. Though, SQ20B/CSCs/EGFR^Low^ population appears resistant to cetuximab, and can explain the recurrence rates [6], and Bonner's hypothesis of a resistant subpopulation in HNSCC [5, 31].

### EMT, invasion/migration and radiation enhancement

Activation of migration by photon radiation has been previously reported in the literature, a result explained by EGFR activation [13, 33, 39]. In most of cases, the receptor is activated with a ligand, wherein EGF is the major one. After photon radiation, EGFR is activated without natural ligand but by cellular stress induced by radiation [40]. In our study, if migration is low in SQ20B parental cell line, it is significantly enhanced by photon radiation (Figure 4). In parallel, photon radiation enhances invasion in both populations. This radiation enhancement could be linked with intracellular signaling pathways activation, causing the secretion of matrix metalloproteases (MMP), particularly MMP-9 [41]. Moreover, cetuximab effectively inhibits migration and invasion in SQ20B cells, which is consistent with literature [42]. Migratory capacities are linked with the EMT phenotype where parental SQ20B cell line presents an epithelial phenotype, and the SQ20B/CSCs behaves mesenchymal. If SQ20B population strongly expresses E-cadherin, an epithelial marker [43], the subpopulation of CSCs loses the expression of E-cadherin in favor of N-cadherin, the mesenchymal marker (Figure 3). It is therefore possible to isolate here a subpopulation of cells from the same line, having all the characteristics of cells with high migratory and invasive potential, to explain the metastatic risk. Such phenotypic characteristics are close to literature, where CSCs present a mesenchymal phenotype [44].

### Radiobiological efficacy of carbon-therapy

High linear energy transfer (LET) radiation has a two times higher RBE than photons [45]. In our study, Carbon ion irradiation increases both SQ20B and SQ20B/CSCs clonogenic cell death (Figure 5), and decreased CSCs proliferation, as reported in the literature in other locations [46]. Carbon beam induce DNA damages in hypoxic area, and thus inhibits CSCs proliferation and survival, in hypoxic niches [47]. In a recent study, carbon-ions appear to induce CSCs apoptosis in a glioblastoma cell line, through prolonged upregulation of phosphorylated p53 [48]. In parallel, carbon-ion irradiation induces apoptosis-related Cytochrome C of triple negative breast CSCs [49]. In a pancreas cell line, heavy-ion irradiation induces irreparable clustered double strand breaks [50]. Data from clonogenic assays performed in our study showed a linear response with increasing doses with a unique alpha component. It is thus a potential weapon against HNSCC CSCs [51]. These results reflect the clinical efficacy of carbon ion in HNSCC patients, reported in several studies [7, 8]. Cetuximab strongly inhibits SQ20B clonogenicity to such an extent that no clones could be isolated and counted to establish the curve with this combination therapy. Caution might be given with this possible therapeutic association knowing the toxicities of cetuximab in combination with photon radiation [31]. However, no supplementary effect of cetuximab associated with the irradiation was observed in SQ20B/CSCs, still linked with the low EGFR expression. In parallel, no data is found on proton therapy and cetuximab efficacy.

In our study, irradiation with high LET reduces migration and invasion in both populations SQ20B and SQ20B/CSCs (Figure 6) in contrast to that obtained after photon irradiation. Hadrontherapy was found to decrease migration and invasion in several studies [13, 52], but no data exists in HNSCC location. Ogata T et al. were the first to introduce heavy ion species in reduction of cell motility [53]. Carbon ion irradiation would block cell motility pathways, decreasing integrins' secretion [54], particularly MMP-2 secretion [52], or Akt phosphorylation [55] in contrast to photon irradiation. In a medulloblastoma cell line, Rieken S el al. have recently demonstrated that heavy-ions could inhibit cell migration through downregulation of MMP-9 and upregulation of proadhesive cell surface integrin alpha-5 leading to increased cell adherence to extracellular matrix proteins [56]. Carbon ion is also known to reduce levels of GTP-bound Rac1 and RhoA, two important regulators of cell motility [57]. EGFR downstream signaling seems to be targeted by Carbon Ion in Lung Cancer [58], as in our study. Cetuximab also inhibits migration and invasion in SQ20B population and with a synergistic activity in combination with carbon ions. This synergistic effect is curiously found significant only on the invasion phenomenon in CSCs population. Accessory pathways such as HER2 and HER3 may play a role in this situation, as it has been hypothesized in some studies [59].

### Emerging new therapeutic modality for HNSCC

To conclude, we isolated here a subpopulation of head and neck cancer stem cells characterized by high migratory and invasive capacities and an insensibility to cetuximab, which could explain local and distant recurrences in HNSCC after treatment. This current therapeutic modality (concomitant cetuximab with photon radiation) effectively targets most of the cells (SQ20B), without impacting the 1% of SQ20B/CSCs intrinsic population. The low EGFR expression in CSCs population explains the inefficiency of cetuximab on these clones, which probably remain after a radiotherapy treatment and cannot be detected with current diagnosis techniques. In this context, hadrontherapy appears to be a very promising therapeutic modality in HNSCC, counteracting migration/invasion process in both parental cells and CSCs in contrast to photon irradiation.

## MATERIALS AND METHODS

### Cell culture

HNSCC SQ20B cell line was derived from a recurrent laryngeal cancer (Gift of John B. Little, Boston, Massachusetts, USA). This cell line is p53 mutated and HPV-negative. CSCs (SQ20B/CSCs), have been generated as previously described [51]. Successive cell sorting have been done to select SQ20B/CSCs from SQ20B parental population using Side Population (SP) through Hoechst exclusion, CD44^High^ and ALDH^High^. These SQ20B parental cells and the associated SQ20B/CSCs were maintained in the same conditions than previously described [51, 60], less than twenty passages for the parental line, and four passages for the SQ20B/CSCs.

### Irradiation

Photon irradiation has been performed with an X-RAD320 irradiator (PrecisionX-ray Inc., NorthBranford, USA), at the Lyon-Sud University (UMS2444/US8 platform, France). The irradiation dose was 2 Gy for migration and invasion assays, as it's usually the case in the clinical practice. Cells have been irradiated at 1, 2, 3, 4 and 5 Gy for the clonogenic cell survival assay at a dose rate of 2 Gy/min.

Carbon ion irradiation (75MeV/n) was performed at the Grand Accélérateur National d'Ions Lourds (GANIL, Caen, France) (Linear Energy Transfer LET = 33.6 keV/μm), as previously described [45, 60]. In previous study, the relative biologic effectiveness (RBE) was about 2 for SQ20B cells [45]. As Mizoe used a fractionation of 4 GyE in his phase II trial [8], we irradiated the cells at 2 Gy physical dose so as to mimic the clinical condition.

### Cetuximab

Cetuximab (C-225, Merck Serono, Darmstadt, Germany) was gently provided by the Pharmaceutical Department of the Institut de Cancérologie Lucien Neuwirth (St Etienne, France), and Centre Hospitalier Universitaire Lyon-Sud (Pierre Bénite, France).

Cell proliferation was checked using the crystal violet method, which allowed determining the half maximal inhibitory concentration (IC_50_) of the association cetuximab. Cells have been maintained for 72 h in 96-wells plates at a density of 10000 cells and then treated with a concentration range of cetuximab from 0.5 nM to 0.5 μM. Cells have been fixed with 1.1% glutaraldehyde and stained with a “crystal violet-in HEPES 20 mM” solution. Cells were lysed with 10% acid acetic and the absorbance has been measured at 550 nm. The assay was conducted in triplicate. Cetuximab (C-225) 5 nM was added to the culture medium 1 h before the irradiation.

### Clonogenic survival assay

Following irradiation, cell survival was carried out using a colony-forming assay as previously described [45, 60]. Briefly, cells were seeded in 25 cm^2^ flasks at different densities 16 h before irradiation, depending on the radiation dose. Then cells were irradiated, and after six cell divisions, colonies were fixed with ethanol 96% and stained with Giemsa 1/20. The number of colonies containing at least more than 64 cells was counted using a Coltcount (Optronix, United Kingdom). The surviving fraction after each treatment was normalized to the surviving fraction for the corresponding control (plating efficiency) and survival curves were fitted using the linear quadratic model following photon radiation or a linear model after carbon ion radiation [61]. The survival fraction at 2 Gy, named SF2, was measured without and with cetuximab for SQ20B and SQ20B/CSCs cells. All experiments have been done at least in triplicate.

### Cell viability and proliferation

Method 1: Cell viability was assessed by Cell Counting Kit-8 (CCK-8 – Sigma-Aldrich, St Louis, USA). Cells were seeded at 10000 cells per well, 3 wells per condition, in 96-well plates and further incubated for 8 h. Then, media were washed and replaced with medium containing or not, cetuximab 5 nM. One hour later, plates were irradiated at 2 Gy. CCK8 reagent was added to each well at 1 h before the endpoint of incubation and optical densities (OD) measured at 450 nm and 650 nm. Experiments have been repeated two times, in triplicate.

Method 2: The xCELLigence RTCA DP system (Ozyme, St Quentin-en-Yvelines, France) was used to monitor cell index. Cells were seeded in 25 cm^2^ flasks for about 16 h. Media was removed and medium containing cetuximab 5 nM was added 1 h before radiation at 2 Gy. Then, cells were harvested and transferred to E-plates at a density of 5000 cells per well, in medium with or without cetuximab 5 nM. The cell index was recorded during 4 days in order to measure doubling time.

### Migration and invasion assays

Migration was performed with a 24-well Transwell chamber with a pore size of 8 μm (Becton Dickinson^®^, Becton Dickinson and Company (BD) New Jersey, USA). Corning^®^ BioCoat™ Growth Factor Reduced BD Matrigel was used according to the manufacturer's instructions to analyze cell invasion. Cells were cultured in 25 cm^2^ flasks for 24 h at a density of 6 × 10^5^ cells per flask. Cells were starved for 24 h with a medium containing 0,1% Bovine Serum Albumine (BSA) instead of FBS, with or without cetuximab 5 nM. After the 2 Gy irradiation, cells were immediately trypsinized and transferred to the upper chamber at a density of 3 × 10^4^ cells. The lower chamber was filled with medium containing 10% FBS as chemoattractant with or without cetuximab 5 nM. After a 24 h incubation (37°C), inserts were fixed and stained with the RAL 555 kit (VWR, Fontenay-sous-Bois, France) and the number of migrating cells was counted. Each assay was performed in triplicate and repeated two times.

### Protein analysis

Cell pellets were lysed in 50 mM Tris buffer (pH 8.0), 150 mM NaCl, 1% Triton X-100, protease inhibitors (Complete Mini, Roche) and anti-phosphatases (PhosSTOP, Roche), for 1 h at 4°C. Lysates were centrifuged for 20 min at 15000 g at 4°C. Protein expression studies were performed by WES, an automated capillary-based size sorting system (ProteinSimple, San Jose CA, USA) [62]. Diluted protein lysate was inserted in plate at a concentration of 0.2 mg/mL. Data was analyzed using Compass software (ProteinSimple, San Jose CA, USA). Primary antibodies used were EGFR (Dilution 1/50) (sc-03; Santa Cruz Biotechnology, Santa Cruz, CA, USA), Phospho-EGFR (Dilution 1/50) (Tyr^1068^; Cell Signaling Technology, Danvers, MA, USA) E Cadherin (Dilution 1/200) (Becton Dickinson Company BD Biosciences, Franklin Lakes, NJ, USA), N Cadherin (Dilution 1/50) (BD Biosciences), Phospho-AKT (Dilution 1/50) (Ser473, Cell Signaling Technology), Phospho-MEK1/2 (Dilution 1/50) (Ser217/221, Cell Signaling Technology) and secondary antibodies were Alpha-Tubulin (Dilution 1/200) and GAPDH (Dilution 1/1000) (Both Santa Cruz Biotechnology). Every protein analysis was performed in triplicate.

### Statistical analysis

Results are expressed as the mean ± Standard Deviation (SD). The differences in means of groups were determined by the Wilcoxon's test, while differences in survival fraction of irradiated cells were analyzed by the Fisher's exact test. The minimum level of significance set at *p* < 0.05.
